# Unveiling the Molecular Origin of Vapor-Liquid Phase Transition of Bulk and Confined Fluids

**DOI:** 10.3390/molecules27092656

**Published:** 2022-04-20

**Authors:** Sorrasit Jitmitsumphan, Tirayoot Sripetdee, Tharathep Chaimueangchuen, Htet Myet Tun, Sorayot Chinkanjanarot, Nikom Klomkliang, Sira Srinives, Woranart Jonglertjunya, Tau Chuan Ling, Poomiwat Phadungbut

**Affiliations:** 1 Nanocomposite Engineering Laboratory (NanoCEN), Department of Chemical Engineering, Faculty of Engineering, Mahidol University, Nakhon Pathom 73170, Thailand; sorrasit.jit@student.mahidol.edu (S.J.); tirayoot.sri@student.mahidol.edu (T.S.); tharathep.cha@student.mahidol.edu (T.C.); htet.tun@student.mahidol.edu (H.M.T.); sira.sri@mahidol.edu (S.S.); 2 National Metal and Materials Technology Center (MTEC), National Science and Technology Development Agency (NSTDA), Pathum Thani 12120, Thailand; sorayot.chi@mtec.or.th; 3 School of Chemical Engineering, Institute of Engineering, Suranaree University of Technology, Nakhon Ratchasima 30000, Thailand; nikom.klo@sut.ac.th; 4 Fermentation Technology Laboratory (FerTechLab), Department of Chemical Engineering, Faculty of Engineering, Mahidol University, Nakhon Pathom 73170, Thailand; woranart.jon@mahidol.edu; 5 Institute of Biological Sciences, Faculty of Science, University of Malaya, Kuala Lumpur 50603, Malaysia; tcling@um.edu.my

**Keywords:** phase transition, Monte Carlo simulation, adsorption, bulk phase, confinement

## Abstract

At temperatures below the critical temperature, discontinuities in the isotherms are one critical issue in the design and construction of separation units, affecting the level of confidence for a prediction of vapor–liquid equilibriums and phase transitions. In this work, we study the molecular mechanisms of fluids that involve the vapor–liquid phase transition in bulk and confinement, utilizing grand canonical (GCE) and meso-canonical (MCE) ensembles of the Monte Carlo simulation. Different geometries of the mesopores, including slit, cylindrical, and spherical, were studied. During phase transitions, condensation/evaporation hysteretic isotherms can be detected by GCE simulation, whereas employing MCE simulation allows us to investigate van der Waals (vdW) loop with a vapor spinodal point, intermediate states, and a liquid spinodal point in the isotherms. Depending on the system, the size of the simulation box, and the MCE method, we are able to identify three distinct groups of vdW-type isotherms for the first time: (1) a smooth S-shaped loop, (2) a stepwise S-shaped loop, and (3) a stepwise S-shaped loop with just a vertical segment. The first isotherm type is noticed in the bulk and pores having small box sizes, in which vapor and liquid phases are close and not clearly identified. The second and the third types occurred in the bulk, cylindrical, and slit mesopores with sufficiently large spaces, where vapor and liquid phases are distinctly separated. Results from our studies provide an insight analysis into vapor–liquid phase transitions, elucidating the effect of the confinement of fluid behaviors in a visual manner.

## 1. Introduction

Fundamental study of fluid phase equilibria is of major significance in many fields of sciences and engineering for the development and optimization of separation processes [1]. Phase diagrams [2], which can have complicated topologies, are frequently used to depict these equilibria. With regard to the modern classification of phase transitions [3], there are two categories by justifying from the first and second derivatives of thermodynamic free energy with respect to chemical potential. The first-order phase transition [4] shows drastic change in the first derivative. This could be attributed to the latent heat, as a result of phase change at a constant temperature. The second-order transition is subtly different [5] since all first derivatives are continuous, while second derivatives have a discontinuity. Such transition could involve the paramagnetic/ferromagnetic transition or the vapor–liquid phase transition at the critical point. In this research, we would focus on the first-order transition, and focus only on the phase transition of vapor and liquid systems.

A system is considered to be in thermodynamic equilibrium when it is mechanically, thermally, and chemically balanced. According to the Gibbs Phase Rule [6,7], the volumetric and thermodynamic properties of a single-phase unary bulk system can be specified by any two intensive independent properties. On the other hand, only one intensive property is required for a two-phase unary bulk system. By this reason, there are many researchers proposing a variety of equations of state (EoS) into the all-in-one framework for robust and time-efficient computations. The simplest basic equation of state is the ideal gas law, applied to rarefied gas with no intermolecular interactions. The well-known cubic equations of state [8,9], i.e., van der Waals (vdW) and modified Soave Redlich Kwong (SRK) equations, are extended to correlate and predict the phase equilibrium properties of fluids for both single- and two-phase systems. In particular, the generic cubic equations of state generate good computational results for the unstable vdW loop of the subcritical vapor–liquid phase transition, owing to the extra degree of freedom. As a result, the Maxwell’s construction of equal areas [10] is applied in searching the tie-line of coexisting phases in order to estimate the saturation vapor pressure and thermodynamic properties of vaporization of a pure substance at a given temperature. It is widely acknowledged that no one EoS can adequately describe all components or fluids, nor can it be used in all conditions. Moreover, the physical understanding of the vdW loop is still incomplete.

Porous materials have been considered as a potent candidate in molecular separation, catalyst support, and medical technology due to the favorable combination of porous characteristics and surface substrates [11,12]. Characteristics of fluids in a confined space, such as pores of a porous media, deviate significantly from those in a bulk state. Besides, geometric shape and the size of a pore dimension leads to a unique microscopic phenomenon that can affect macroscopic situations inside a pore. Classical tools for the characterization of a porous material involve experimental apparatus for an adsorption/desorption test, in which a noble gas, such as nitrogen or argon, can be used as a standard probe [13]. Notably, vapor–liquid phase transitions in the confined media can be monitored when the adsorption/desorption hysteresis exists in the experimental and simulated adsorption isotherms [14]. The width of the hysteresis loop depends on the temperature, pore size, and surface affinity of a porous material [15,16]. For a certain pore size, the hysteresis loop will disappear when the temperature is above the critical hysteresis temperature. Likewise, for a given temperature, the absence of hysteresis will be observed in pores narrower than a critical hysteresis pore size. In summary, compared to the bulk system, the dependence of pore confinement contributes to the vapor–liquid phase transition accompanied by condensation/evaporation hysteresis at a pressure lower than the saturation bulk pressure and at a temperature less than the critical temperature of bulk fluids [17]. After completing the capillary condensation, the fluid in the pore is in the state of liquid-like with a higher density than the bulk system. To gain a better understanding regarding the pore texture inside the disordered materials, where the hysteresis loop is not symmetrical, the scanning curves [18,19,20] can alternatively be obtained by starting from any given point placed at the boundary of the hysteresis loop, and increasing or decreasing the pressures over another boundary. The sub-hysteresis loop could be obtained nested inside the main loop, in which a new pore domain is discovered. However, the hysteresis loop and sub-loops determination is still not adequate for an explanation of a microscopic mechanism of phase transition inside the pores.

Despite the fact that a tremendous database of phase equilibria in bulk and adsorption systems for a single component has been generated by a large number of experiments and simulations [21,22,23,24,25,26,27,28,29], the molecular mechanisms underlying the vapor–liquid transition still remain obscure. In the current work, we employ Monte Carlo simulations in a combination of grand canonical and meso-canonical ensembles, which allow us to deeply investigate the microscopic configurations of fluids in bulk and confined adsorption spaces: slit, cylindrical, and spherical mesopores. We also propose a classification for the various types of vdW isotherms according to their molecular characteristics.

## 2. Results and Discussion

In Section 2.1, Section 2.2, Section 2.3 and Section 2.4, we first use the 1-LJ spherical model of nitrogen to explore the molecular behaviors of the vapor–liquid phase transition at 77 K, followed by comparing those results with the 2-LJ model (Section 2.5). The selected temperature is equivalent to the normal boiling point and below the critical point of bulk nitrogen. Sets of computational scenarios are presented in Table 1. By analyzing the molecular mechanisms of the vapor–liquid phase transition, we have initially categorized the MCE isotherms into the three groups and have summarized what we observed in the fourth column of Table 1. 

### 2.1. Vapor–Liquid Phase Transition of Nitrogen in the Bulk Phase

**Figure 1:**
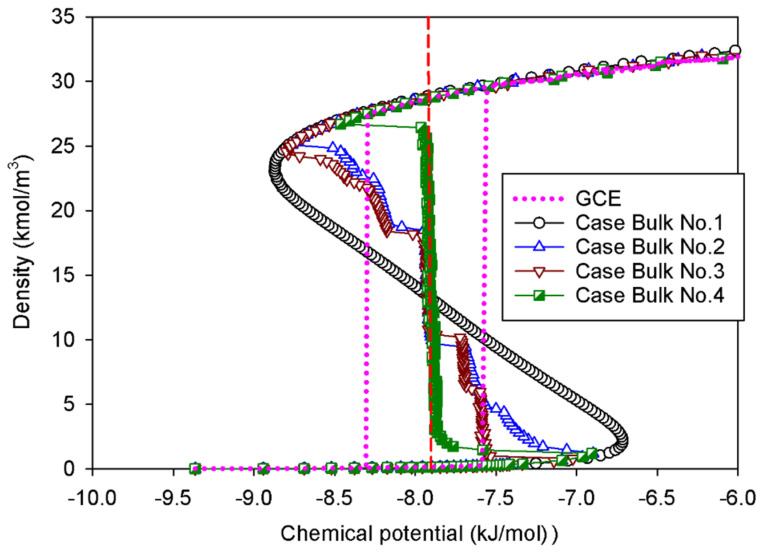
GCE and MCE isotherms of bulk nitrogen at 77 K in four different cases. The red vertical dashed line represents the vapor–liquid phase coexistence at chemical potential of −7.88 kJ/mol.

The GCE (open system) and MCE (closed system) isotherms of bulk nitrogen at 77 K are presented in Figure 1. As physically expected, we observe a good agreement between the GCE and MCE isotherms in the region of the stable state. The GCE isotherm displays the prominent hysteresis loop with a discontinuous change, indicative of a sign of the first-order phase transition [4]. A closed-loop hysteresis is most commonly caused by different metastable states of fluids in which there are abrupt condensation and evaporation at chemical potentials above and below the vapor–liquid coexistence chemical potential (*μ_e_*), respectively. As a result, the GCE isotherm provides no information on how the phase evolves across the transition due to the energy fluctuation, which allows the system to overcome the free energy barrier of two-phase separation.

In the MCE simulations, their isotherms exhibit unstable vdW loops with the vapor and liquid spinodal points. These points represent the thermodynamic limits of metastability. The regions with increasing chemical potential in relation to the density are either stable or metastable, whereas the regions with decreasing chemical potential are unstable. Surprisingly, the density of the system is not affected by the simulation ensembles in the stable regions of rarefied and dense phases. An increase in density causes a drop in chemical potential at the intermediate state, where the liquid expands and the gas space contracts. The MCE isotherm exhibits a smooth vdW loop with an S-shape for Bulk No.1, while others present the isotherms with vertical segments. 

**Figure 2:**
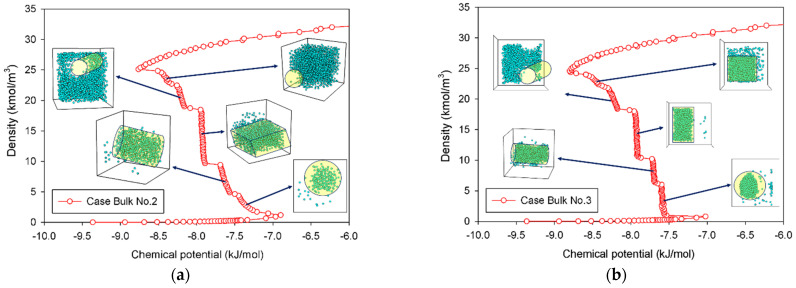

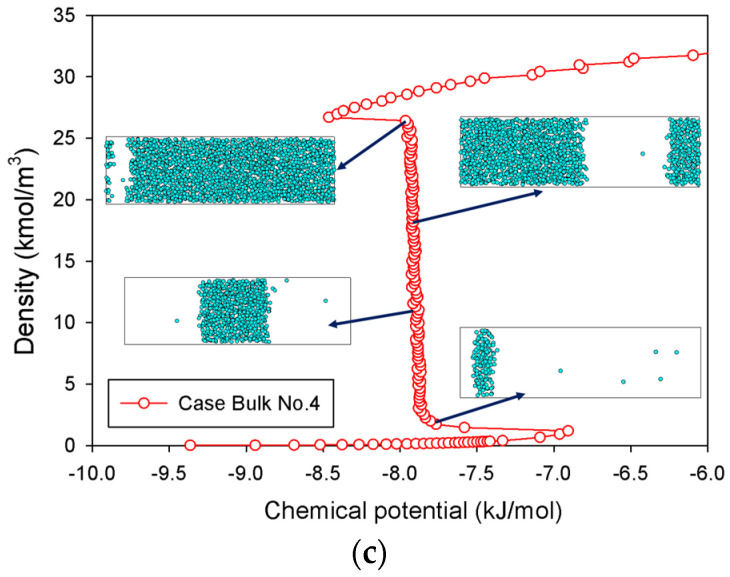
Bulk phase isotherms of bulk nitrogen and their molecular configurations for scenarios of (**a**) Bulk No.2, (**b**) Bulk No.3, and (**c**) Bulk No.4.

The MCE isotherms of nitrogen at 77 K together with molecular snapshots at various points across the isotherm highlight phase transition mechanisms for four different scenarios (Table 1) in Figure 2a–c. In this range, we can categorize the MCE isotherms into three groups. Based on each group, the relationships between the isotherms of bulk nitrogen and their molecular configurations will be discussed. 

### 2.1.1. Group I: Smooth S-Shaped Isotherm

Group I represents the smooth S-shaped isotherm found in Bulk No.1, in which the unit cell is very small (white circles in Figure 1). This is a classical vdW-type isotherm commonly established in both classical and statistical thermodynamics. By means of molecular simulation, it is observed when the box dimension of bulk fluid is very small [30]. The nucleation of the liquid nucleus can hardly form in a compact area, resulting in a vapor spinodal point occurring at a higher chemical potential as compared to other scenarios. Likewise, the liquid spinodal point exhibits at a lower chemical potential due to the invisible bubble formation. As a consequence, we can only observe the system’s uniform state with the addition of molecules during the vapor–liquid phase transition along the isotherm. 

In order to determine the equilibrium phase transition from this isotherm group, this allows us to additionally compute the coexistence chemical potential using Maxwell’s rule of equal areas along the isotherm [31]:

(1) $$\oint_{\mu_{e}}\rho \left(\mu \right)\;d\mu =0$$

Noticeably, the positions of the clear vertical segments of the isotherms and equilibrium phase coexistence between the vapor and liquid phases in all scenarios, as illustrated by the red dashed line in Figure 1 at the identical chemical potential of −7.88 kJ/mol. To support our conjecture, the obtained coexistence chemical potential agree well with the computational results, reported by the kinetic Monte Carlo simulation [32], and that of the Johnson equation of state [33], which states that the vapor–liquid equilibrium in the bulk 1-LJ fluid at *k_B_T/ε_FF_* = 0.762 (or 77 K) is *μ_e_* = −9.34*ε_FF_* (or −7.88 kJ/mol). This suggests that the equilibrium thermodynamic properties of bulk fluids are not significantly affected by the sizes of simulation box.

#### 2.1.2. Group II: Stepwise S-Shaped Isotherm

In the case of Bulk No.2 and No.3 (Figure 2a,b), their isotherms follow Group II, in which vapor and liquid phases can be segregated when the dimension of the cubic box becomes larger. From the vapor spinodal point, we can see the first nucleation of a spherical liquid droplet with the convex interface clustering in the system. Since there is adequate space to form the liquid droplet in order to balance enthalpic and entropic effects, the vapor spinodal point occurs at a lower chemical potential than the Bulk No.1 isotherm. As the nitrogen loading is increased (for Bulk No.2) or the box volume becomes compacted (for Bulk No.3), the spherical droplet grows bigger, and eventually transforms into a cylindrical droplet, occupying a less convex interface. The vertical segments corresponding to the coexistent chemical potential (*μ_e_*) are then met when the fluid density in the box further rises. The liquid with two planar interfaces is formed at this stage. After this, the rarefied phase shrinks in size and then merges with the surrounding liquid to form a cylindrical bubble with the concave interface. On further addition of density, the spherical bubble with a more concave interface appears and then decreases in size at the liquid spinodal point. Therefore, the higher degree of concavity of the interface, the lower the chemical potential in the unstable intermediate state. Note that the critical sizes of a liquid droplet near the vapor spinodal point and a vapor bubble near the liquid spinodal point correlate to operating temperature and size of the simulation box. 

However, we can see distinguishable molecular characteristics in these two scenarios. The formation of both spherical and cylindrical liquid droplets for Bulk No.2 occurs as the pressure is gradually reduced by supplying molecules from the surrounding rarefied phase, whereas those for Bulk No.3 occur as the chemical potential in the dosing cell is maintained, allowing the box volume to control the geometrical shape of liquid condensate. Based on our knowledge, these distinct behaviors of the bulk fluids have never been reported in the literature.

#### 2.1.3. Group III: Stepwise S-Shaped Isotherm with Just a Vertical Segment

A long and clear vertical segment representing the two-phase coexistence in an isotherm represents a phenomenon in a rectangular pore (Figure 2c). In lieu of a spherical liquid droplet, the first nucleation of the thin slab liquid with two planar interfaces can only be formed in the intermediate state, followed by the expansion of the liquid phase as an increase in loading. This process is similar to the one described in Group II, except that the only vertical section is now located at the coexisting chemical potential. When the rectangular bubble reaches a critical size, it then dissipates at the liquid spinodal point.

**Figure 3:**
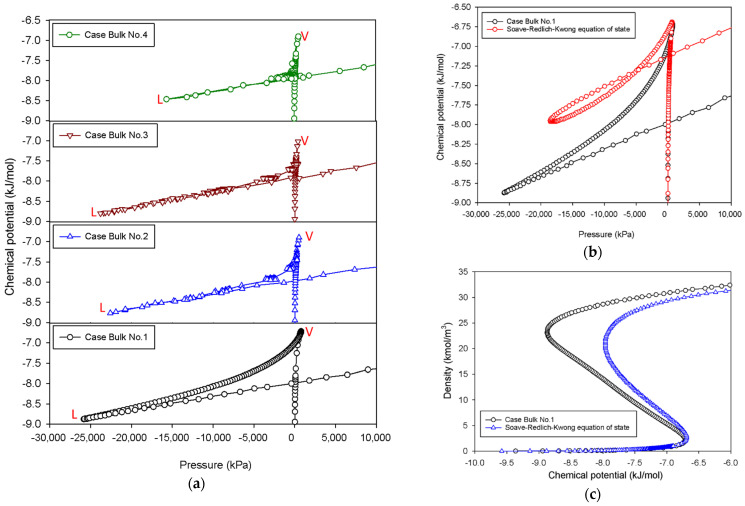
(**a**) Chemical potential vs pressure for the bulk phase transition of nitrogen at 77 K for four different scenarios. “V” and “L” stand for vapor and liquid spinodal points, respectively. (**b**) Chemical potential vs pressure for the bulk phase transition of nitrogen at 77 K for Bulk No.1 and that for Soave–Redlich–Kwong equation of state. (**c**) Bulk nitrogen isotherms of Bulk No.1 and Soave–Redlich–Kwong equation of state.

Figure 3a shows the thermodynamic relationship between the chemical potential and the pressure of nitrogen at 77 K for various scenarios. The plots depict an unstable triangle-shaped zone with different areas. The triangular area of the *μ*-*P* plot is the greatest for the case of Bulk No.1 when compared to all MCE isotherm groups; this is owing to the continuous decline in chemical potential in the intermediate stage. Our obtained results are a typical feature of vdW fluids [34], and they qualitatively coincide with cubic equations of state such as the Soave–Redlich–Kwong equation of state [35], as shown in Figure 3b,c. When the dimension of the simulation box is adjusted so that the two-phase separations are visible during the transition, the triangular parts of *μ*-*P* plot substantially drop because of the existence of vertical segments in the MCE isotherms.

As inferred from three different groups of MCE isotherms examined in bulk nitrogen, these will be used as a basis for our subsequent discussion of the phase transition when the degree of confinement is higher.

### 2.2. Nitrogen Adsorption in the Infinite Slit Mesopore

**Figure 4:**
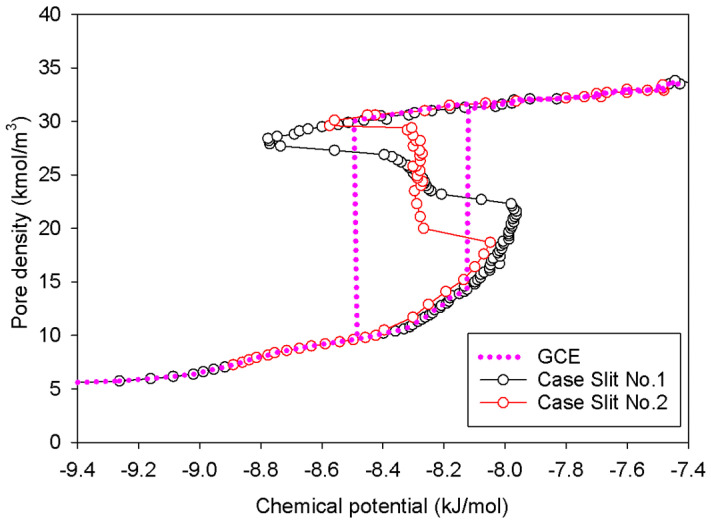
GCE and MCE nitrogen adsorption isotherms in the infinite slit pore of 5 nm width at 77 K.

Figure 4 shows the GCE and MCE isotherms for examining the vapor–liquid phase transition of nitrogen at 77 K using a graphitic slit pore of 5 nm width with infinite extent in the *x*- and *y*-directions. For both simulations, the adsorption begins with the molecular layering of adsorbate on the two opposite graphitic surfaces and finishes with the densification of the condensed adsorbate; these are counted as the stable states. Analogous to the bulk phase system, the hysteresis loop of Type H1 is obviously found by the GCE simulation, indicative of the existence of phase transition. Along the adsorption and desorption branch, the states of the adsorbed layers and condensed phase shift from stable to metastable. The process of rapid condensation occurs when the two undulating interfaces from the two opposite adsorbed layers at the metastable state join together due to the effect of thermal motion at the mass transfer zone [36]. In desorption, the instant evaporation proceeds by starting from the fully condensed phase, followed by the stretching and cavitation of the condensate resulting in the stable adsorbed layers only remaining in the pore. As a result, the metastable adsorbed layers just prior to condensation are thicker and denser than the stable layers just after evaporation.

MCE simulation, on the other hand, investigates two distinct scenarios (Slits No.1 and No.2 as noted in Table 1) based on the surface dimension of pore walls. As indicated in the preceding section, the MCE isotherm for Slit No.1 is comparable to Group I, but Group III corresponds to the isotherm of Slit No.2. The vapor–liquid phase coexistence can be calculated using the Maxwell’s construction of equal areas along the isotherm of Slit No.1 or by tracing the vertical segment in the isotherm of Slit No.2. Overall, the coexistence chemical potential (or pressure) for both scenarios is the same: −8.31 kJ/mol. Two scenarios are then chosen to study their graphics at the molecular level in order to give further light on how the phase evolves in the infinite slit pore.

**Figure 5:**
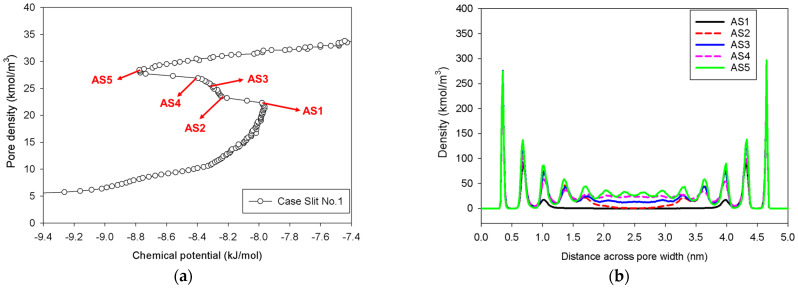

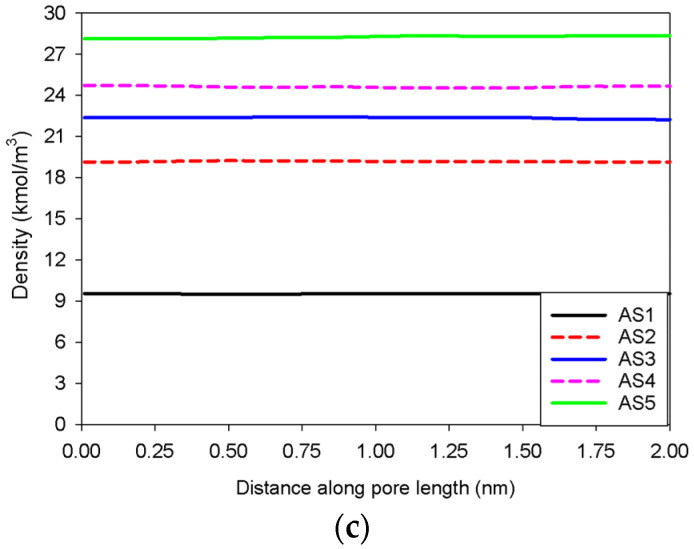
(**a**) Nitrogen adsorption isotherm in the infinite slit pore of 5 nm width at 77 K for Slit No.1, and its local density distributions (**b**) across pore width and (**c**) along pore length.

Figure 5 illustrates the MCE simulation results as well as local analyses along pore width and pore length of various points marked in Figure 5a for the infinite slit pore with a pore wall that is 2 nm long in the *y*-direction (denoted as Slit No.1). The density distribution along the pore length consistently rises with increasing loading from vapor to liquid spinodal points (see Points AS1–AS5 in Figure 5c), while the adsorbed layers increasingly thicken (see Points AS1–AS5 in Figure 5b) from three to seven layers from adjacent pore walls. As a result, apart from the growth of the adsorbed layers and the shrinkage of the gas-like core, we are unable to identify any clear phase segregation in the unstable intermediate state.

**Figure 6:**
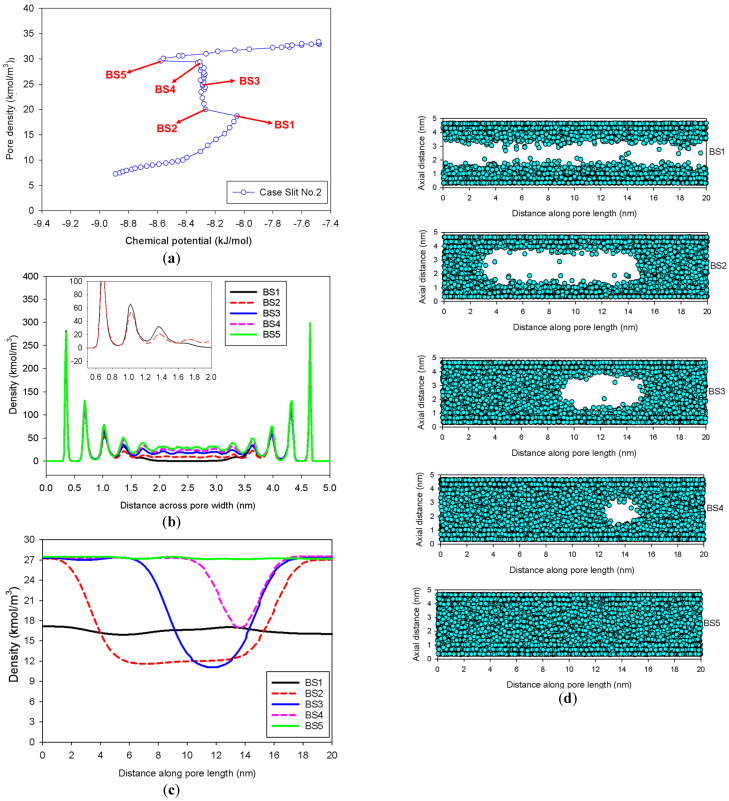
(**a**) Nitrogen adsorption isotherm in the infinite slit pore of 5 nm width at 77 K for Slit No.2, its local density distributions (**b**) across pore width and (**c**) along pore length, and (**d**) molecular snapshots of nitrogen on each point.

Figure 6 displays the MCE isotherms, local densities along the pore width and pore length, and the corresponding molecular snapshots as a function of the distance along the pore length for a slit pore of 5 nm width having an infinite pore length of 20 nm (Slit No.2). At first, from a very low pressure to Point BS1, the adsorbed layers build up on two opposite walls concurrently. There are only two phases observed at this stage: the adsorbed layers and the gas-like core. With a tiny amount of adsorbate, the mechanism shifts from molecular layering to a liquid bridge formation with the concave interface from Points BS1 to BS2, resulting in a three-phase coexistence in the pore: the stable adsorbed layer, the liquid bridge, and the gas-like region (Point BS2 in Figure 6c). However, there are a number of remarkable features that have not been well recognized in the literature:

(1) At Point BS1 of Figure 6c, the threshold density of the metastable adsorbed layers is about 17 kmol/m^3^, where the adsorbed films on opposite pore walls are close enough to create a liquid bridge.
(2) At Point BS2 of Figure 6c, this is the process of nucleation of a liquid bridge at the coexistence chemical potential [37]. The densities of stable adsorbed layers and the liquid embryo are approximately 12 and 27 kmol/m^3^, respectively. The liquid bridge has two concave cylindrical menisci, which indicate the reduction of chemical potential.
(3) The creation of a liquid bridge between Points BS1 and BS2 is mostly due to a decrease in density in the second and third adsorbed layers (as shown in the inset of Figure 6b). This is why the density of stable adsorbed layers is lower than that of the metastable adsorbed layers.
(4) The axial density of the metastable adsorbed layer at Point BS1 is noticeably higher than at Point AS1, indicating that the surface dimension of the carbon substrate for Slit No.1 is insufficient to construct the liquid bridge.
(5) The density at Point BS1 is greater than that at the point just before sudden condensation of the GCE isotherm. This is because the minute size of the dosing cell used in the MCE simulation allows the adsorption system to control a much narrower undulating zone between the adsorbed layers and the gas-like core to be substantially smaller [38], requiring a greater chemical potential to build up the adsorbed layer for condensation.

As loading increases from Point BS2, two concave menisci at the liquid bridge gradually move along the pore length with constant density of the adsorbed layer and constant radii of curvature at the coexistence chemical potential. When the menisci reach Point BS4, we can observe that the lowest density of the gas-like core is the same as the density of the metastable adsorbed layer (17 kmol/m^3^), as illustrated in Figure 6c. This has never been documented in the literature regarding the critical density for bubble collapse.

Then, a further increase in loading results in the gas-like bubble at Point B5, the liquid spinodal point, disappearing. Interestingly, the average density at Point BS5 is the exact same as that at Point AS5, implying that the condensation along the unstable state is complete. Furthermore, it is worth mentioning that this coexistence chemical potential of the slit is less than that of bulk system due to the biconcave menisci of the liquid bridge conforming to the Cohan theory [39].

### 2.3. Nitrogen Adsorption in the Infinite Cylindrical Mesopore

**Figure 7:**
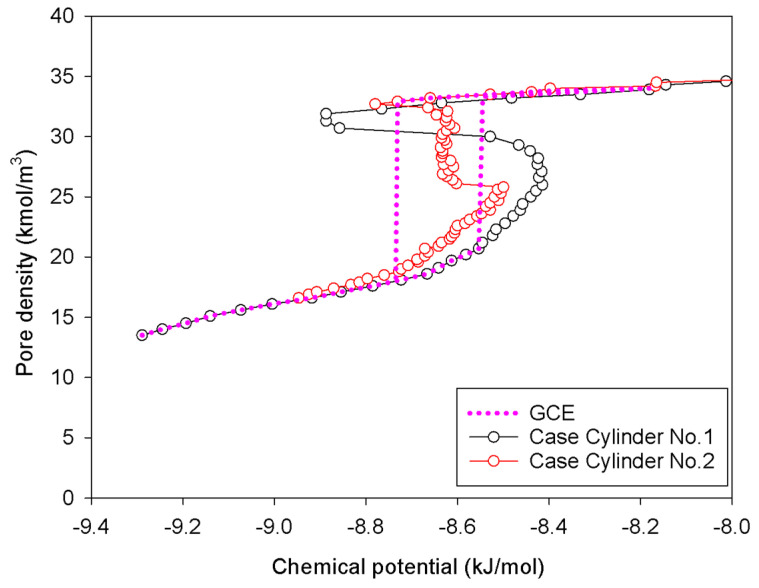
GCE and MCE nitrogen adsorption isotherms in the infinite cylindrical pore of 5 nm in diameter at 77 K.

**Figure 8:**
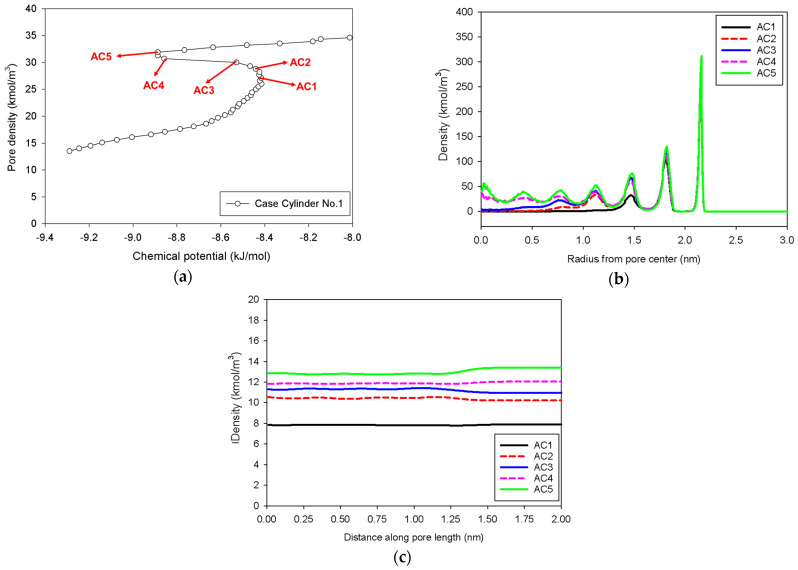
(**a**) Nitrogen adsorption isotherm in the infinite cylindrical pore of 5 nm in diameter at 77 K for Cylinder No.1, and its local density distributions (**b**) across pore radius and (**c**) along pore length.

**Figure 9:**
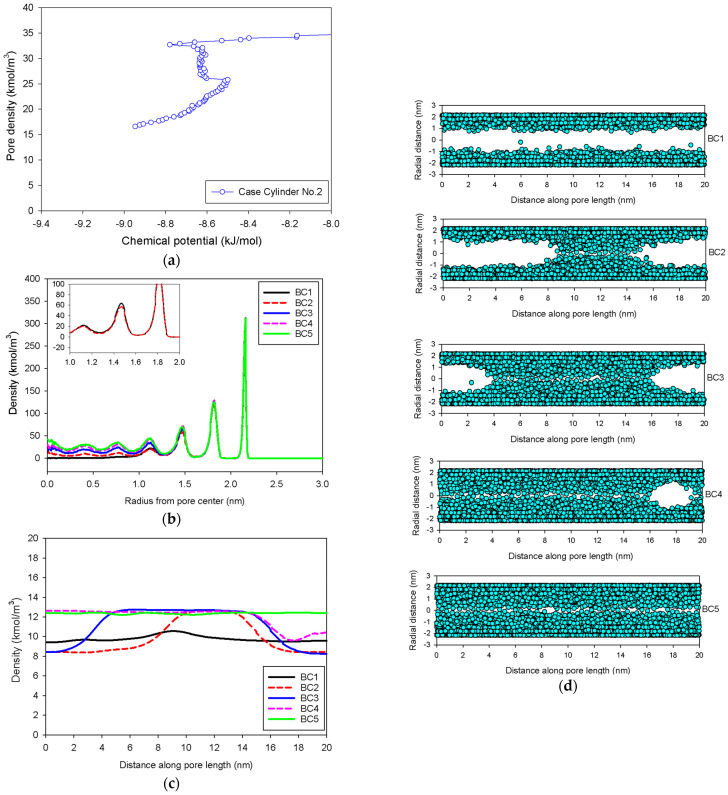
(**a**) Nitrogen adsorption isotherm in the infinite cylindrical pore of 5 nm in diameter at 77 K for Cylinder No.2, its local density distributions (**b**) across pore radius and (**c**) along pore length, and (**d**) molecular snapshots of nitrogen on each point.

Figure 7 shows the GCE and MCE isotherms for nitrogen adsorption at 77 K in an infinite cylindrical pore of 5 nm in diameter at various pore lengths, whereas Figure 8 and Figure 9 depict local density distributions in radial and axial axes.

In general, the isotherms and their adsorption mechanisms appear to be identical to those observed in the slit pores. For instance, the MCE isotherms of Cylinder No.1 and No.2 are classified to be Group I and III, respectively. All observed molecular behaviors in the cylindrical pores via MCE simulation are identical to those postulated by Everett–Haynes [40]. However, due to the influence of confinement, we make the following observations that distinguish the microscopic configurations of adsorbate in the Cylinder No.2 from those in the Slit No.2:

(1) Adsorption progresses up to Point BC1 via the formation of cylindrical interfacial curvature by the accumulation of metastable adsorbed layers across the radial direction (Figure 9b).
(2) The curvature of the interface changes from cylinder to hemispheres during the formation of the liquid bridge by supplying molecules from the second adsorbed layer (Point BC2 in Figure 9b). This differs from what we found in the slit pore, where the interfacial curvature ranges from two parallel slabs to semi-cylindrical menisci.
(3) All spinodal points and the equilibrium phase transition occur at a lower chemical potential than in slit pore; this is attributed to the larger curvature of the interface suppressing the size of the undulating interface [41]. For a given pore size, the condensation in a cylinder requires fewer adsorbed layers than in a slit pore, and the core size in a cylinder just before capillary condensation is greater than in a slit pore.

### 2.4. Nitrogen Adsorption in the Spherical Mesopore

**Figure 10:**
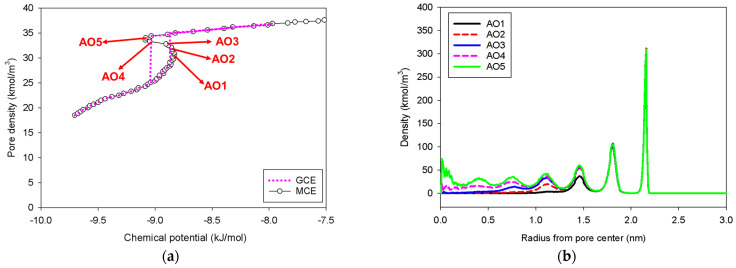
(**a**) Nitrogen adsorption isotherm in the spherical pore of 5 nm in diameter at 77 K, and (**b**) its local density distributions across pore radius.

This pore shape is commonly utilized to study the bubble formation and nucleation barrier within the pore [42]. Figure 10 presents the GCE and MCE isotherms of nitrogen at 77 K in a 5-nm spherical pore, together with the radial density distributions. As discussed in previous sections, the GCE isotherm has a significant hysteresis loop corresponding to spontaneous condensation and evaporation in a metastable state, but the MCE isotherm just has a continuous and smooth S-shaped isotherm with vapor and liquid spinodal points (Group I). Following the evolution of the phase transition starting from low pressure to the vapor spinodal point (Point AO2), there are two phases found at this stage: the two metastable adsorbed layers and the gas-like core. Because the system does not have enough area to support a stable condensed phase once the loading reaches Point AO4, no liquid bridge is identified throughout the transition. As a result, our conclusion is compatible with the examples of Bulk No.1, Slit No.1, and Cylinder No.1.

### 2.5. Effect of Molecular Model of Nitrogen

**Figure 11:**
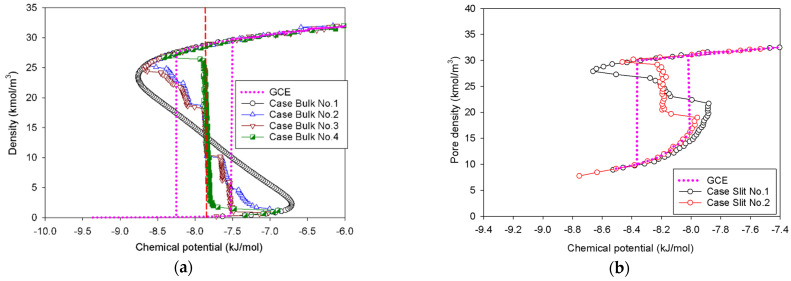

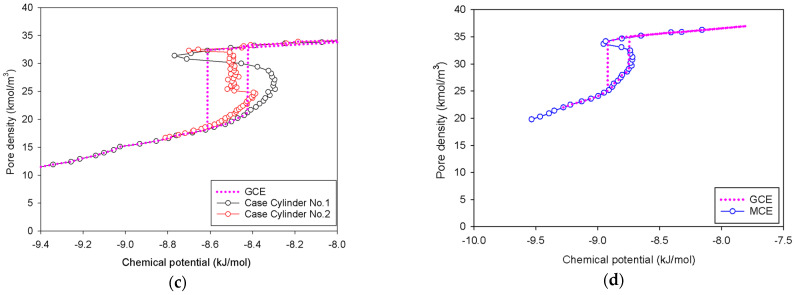
GCE and MCE isotherms of nitrogen with 2-LJ model: (**a**) bulk nitrogen at 77 K in four different cases, and nitrogen adsorption at 77 K in the (**b**) infinite slit pores, (**c**) cylindrical pores, and (**d**) spherical pores.

The effect of molecular elongation of nitrogen molecules between the 1-LJ and 2-LJ models, is also studied for the same scenarios as stated in Table 1 and addressed in Section 2.1, Section 2.2, Section 2.3 and Section 2.4; the GCE and MCE isotherms at various cases are displayed in Figure 11.

We can derive the same conclusions as in the 1-LJ model, with the sole difference being that the coexistence chemical potential of the 2-LJ model in every scenario is greater than that of the 1-LJ model. This implies that the elongation effect of the nitrogen model is independent of the classification of the MCE isotherms and their molecular mechanisms.

## 3. Materials and Methods

### 3.1. Fluid–Fluid Interaction Models

In this molecular simulation study, we have focused on two potential models of nitrogen: (1) the pseudo-spherical model proposed by Ravikovitch et al. [43] (1-LJ model) and (2) the TraPPE model with two Lennard-Jones, and three atomic partial charge sites [44] (2-LJ model). The fluid–fluid interaction was modeled by a combination of the Lennard-Jones (LJ) 12-6 equation and the electrostatic Coulomb potential equation:

(2) $$U_{FF}\left(r_{ij}\right)=-\;4\varepsilon_{FF}\left[{\left(\frac{\sigma_{FF}}{r_{ij}}\right)}^{12}-{\left(\frac{\sigma_{FF}}{r_{ij}}\right)}^{6}\right]+\frac{q_{i}q_{j}}{4\pi \varepsilon_{0}r_{ij}}$$

where *ε_FF_* is the energy well-depth, *σ_FF_* is the collision diameter, *q_i_* and *q_j_* are partial charges of site *i* and *j* respectively, *ε*_0_ is the constant permittivity of free space, and *r_ij_* is the distance between atomic sites *i* and *j*. The molecular parameters of two nitrogen models are summarized in Table 2.

### 3.2. Solid–Fluid Interaction Models

**Figure 12:**
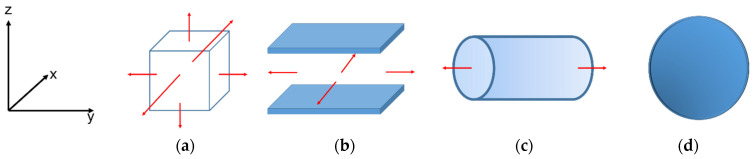
Schematic diagram of the studied systems: (**a**) infinite bulk system, (**b**) infinite slit pore, (**c**) infinite cylindrical pore, and (**d**) spherical pore. The red arrow lines represent the directions of periodic boundary conditions applied.

Bulk geometry was introduced as an infinite unit cell, in which a cubic box represents the bulk. The other three geometries of confined space were computed using idealized carbon pore models with the shape of spherical, infinite cylindrical, and infinite slit pores. All the pores were modeled with a graphitic surface density (*ρ_s_*) of 38.2 nm^−2^, and a spacing between two adjacent graphitic layers (Δ) of 0.3354 nm. The molecular parameters of a carbon atom in a graphene sheet were *σ_SS_* = 0.34 nm and *ε_SS_/k_B_* = 28 K. The cross molecular parameters (*σ_SF_* and *ε_SF_*) between solid–fluid interactions were calculated with the Lorentz–Berthelot mixing rules. Periodic boundary conditions were applied to model infinite-length boundaries with regard to the red arrow lines in Figure 12a–c.

According to the solid–fluid interaction potential, we adopted a 10-4-3 family of interaction models for those pore geometries with a semi-infinite number of graphitic layers [45].

(3) $$U_{SF}=2\pi \rho_{S}\sigma_{SF}^{2}\varepsilon_{SF}\left[U_{SF,1}-U_{SF,2}-U_{SF,3}\right]$$

where *U_SF_*_,1_, *U_SF_*_,2_, and *U_SF_*_,3_ are repulsive, attractive, and continuum solid terms. They are a function of pore geometry as presented in Table 3.

### 3.3. Monte Carlo Simulations

In the Monte Carlo simulation with grand canonical ensemble (GCE) [46], at least 100,000 cycles in both the equilibration and sampling stages were applied. Each cycle consisted of 1000 trials of local displacement, insertion, and deletion of a nitrogen molecule from the bulk phase with equal probability. At a given point on the GCE isotherms, simulation inputs were chemical potential, temperature, and simulation box volume. The cut-off radius for the fluid–fluid potential calculation was set to be half of the largest dimension of the simulation box.

The meso-canonical ensemble (MCE) [47], also known as the gauge cell method [48] was employed to study the vapor–liquid phase transition in bulk and idealized pore systems. There are two simulation boxes: the adsorption and dosing cells. The box dimension of the adsorption cell was exactly the same as that studied in the GCE simulations, while the cubic dimension of the dosing cell was constantly kept at 8 nm, allowing bulk pressure and chemical potential to be calculated using the virial equation and the Widom insertion method, respectively [49,50]. This dosing volume was found to be sufficient for tracing the isolated unstable state [51]. Based on the statistical mechanics theory, the MCE method resembles the GCE method when the size of the dosing cell is infinite, whereas it is identical to the traditional canonical ensemble method when the cell size becomes zero. During the MCE simulation, in order to acquire a point on the isotherm, attempting local displacement within the same box and global displacement between two boxes were the major trial moves with equal probability. We used the exactly same number of cycles and trials as previously mentioned in the GCE simulation.

At the end of the sampling stage for both GCE and MCE simulations, the fluid density within the system is defined as the number of nitrogen molecules per unit volume by the following equation:

(4) $$\rho =\frac{\langle N\rangle }{V}$$

where <*N*> is the ensemble average of the number of nitrogen molecules residing in the system. *V* is the simulation box volume for the bulk–phase system or accessible pore volume for pore systems, defined as the volume in which the solid–fluid potential between a nitrogen molecule and the pore is non-positive [52].

The local density distributions along the pore length, the pore width, and the pore radius were calculated by dividing into differential bins in *y*-, *z*-, and *r*-directions, respectively:

(5) $$\rho \left(x^{\prime }\right)=\frac{\langle \Delta N\left(x^{\prime }\right)\rangle }{\Delta V\left(x^{\prime }\right)}$$

where *x*′ is the selected *y*-, *z*-, or *r*- directions for computing, <Δ*N*(*x*′)> is the ensemble average number of nitrogen molecules in a differential bin bounded by [*x*′, *x*′ + Δ*x*′], whose volume is Δ*V*(*x*′). The spacing for each bin (Δ*x*′) was chosen to be 0.05*σ_FF_*. 

## 4. Conclusions

In this work, we have extensively employed Monte Carlo simulations with grand canonical and meso-canonical ensembles to clarify the molecular behaviors of nitrogen during the transition from rarefied to condensed phases at 77 K in bulk-phase and mesoporous systems. Regarding our computational discoveries, each MCE isotherm has some characteristics in common: vapor and liquid spinodal points, and intermediate states. Nevertheless, the simulated MCE isotherms during the phase transition can be categorized into three groups based on the volume of the simulation box or the lateral dimension of infinite-length pores: 

Group I: the smooth S-shaped isotherm, which applies to the condensation in a small bulk volume, the small surface dimensions of slit and cylindrical pores, and a spherical pore, all of which are linked to the indistinct partitioning of vapor and liquid phases during transition.

Group II: the stepwise S-shaped isotherm, which applies only to a large cubic volume of the bulk phase transition where the spherical droplet, the cylindrical liquid condensate, the liquid slabs, and the cylindrical and spherical bubbles can occur in the system. 

Group III: the stepwise S-shaped isotherm with just a vertical segment, which applies to the condensation in a rectangular bulk volume and slit and/or the cylindrical mesopores with large surface dimensions where only either a liquid bridge or a liquid slab can be found before the condensation process is completed. The liquid bridge is nucleated by taking the molecules from metastable adsorbed layers at the vapor spinodal point. Meanwhile, at the liquid spinodal point, the vapor bubble disappears when the density of the bubble is identical to the density of the metastable adsorbed layers.

## Figures and Tables

**Table 1:** Explanations of different cases for the bulk-phase and pore systems to be modelled by MCE simulation.

Type of System	Case	MCE Simulation Detail	Type of MCE Isotherm
Bulk phase	Bulk No.1	Gradual addition of nitrogen molecules in a square box having a linear dimension of 2 nm.	Group I
	Bulk No.2	Gradual addition of nitrogen molecules in a square box having a linear dimension of 5 nm.	Group II
	Bulk No.3	Placing 1000 nitrogen molecules in a square box with varying box volume.	Group II
	Bulk No.4	Gradual addition of nitrogen molecules in a rectangular box of 2.5 × 20 × 2.5 nm^3^.	Group III
Infinite slit pore of 5 nm in width	Slit No.1	Gradual addition of nitrogen molecules in an infinite slit pore having pore length in *y*-direction of 2 nm.	Group I
	Slit No.2	Gradual addition of nitrogen molecules in an infinite slit pore having pore length in *y*-direction of 20 nm.	Group III
Infinite cylindrical pore of 5 nm in diameter	Cylinder No.1	Gradual addition of nitrogen molecules in an infinite cylindrical pore having pore length in *y*-direction of 2 nm.	Group I
	Cylinder No.2	Gradual addition of nitrogen molecules in an infinite cylindrical pore having pore length in *y*-direction of 20 nm.	Group III
Spherical pore of 5 nm in diameter	Sphere	Gradual addition of nitrogen molecules in a spherical pore.	Group I

**Table 2:** Molecular parameters of nitrogen used in this work.

Potential Model of Nitrogen	Interacting Site	*x* (nm)	*y* (nm)	*z* (nm)	*σ_FF_* (nm)	*ε_FF_/k_B_* (K)	*q* (e)
1-LJ model	N_2_	0	0	0	0.3615	101.5	0
2-LJ model	N	−0.055	0	0	0.331	36.0	−0.482
	Center site	0	0	0	0	0	+0.964
	N	+0.055	0	0	0.331	36.0	−0.482

**Table 3:** Equations of *U_SF_*, *U_SF_*_,1_, *U_SF_*_,2_, and *U_SF_*_,3_ used in Equation (3) based on different pore geometries.

Type of Pore Geometry	Term	Equation
Spherical pore	$U_{SF}$	$U_{SF}=U_{SF}\left(r,R\right)$ where *r* is the radial distance from pore center and *R* is pore radius.
	$U_{SF,1}$	$U_{SF,1}\left(r,R\right)=\frac{2}{5}\sum_{i=0}^{9}\left[\frac{\sigma_{SF}^{10}}{R^{i}{\left(R-r\right)}^{10-i}}+\frac{\sigma_{SF}^{10}}{R^{i}{\left(R+r\right)}^{10-i}}\right]$
	$U_{SF,2}$	$U_{SF,2}\left(r,R\right)=\sum_{i=0}^{3}\left[\frac{\sigma_{SF}^{4}}{R^{i}{\left(R-r\right)}^{4-i}}+\frac{\sigma_{SF}^{4}}{R^{i}{\left(R+r\right)}^{4-i}}\right]$
	$U_{SF,3}$	$U_{SF,3}\left(r,R\right)=\frac{\sigma_{SF}}{3\Delta }\left\{\begin{array}{c} {\left(\frac{\sigma_{SF}}{R+0.61\Delta -r}\right)}^{3}+{\left(\frac{\sigma_{SF}}{R+0.61\Delta +r}\right)}^{3}\\ +\frac{3}{2}\sum_{i=1}^{2}\left[\frac{\sigma_{SF}^{3}}{{\left(R+0.61\Delta \right)}^{i}{\left(R+0.61\Delta -r\right)}^{3-i}}+\frac{\sigma_{SF}^{3}}{{\left(R+0.61\Delta \right)}^{i}{\left(R+0.61\Delta +r\right)}^{3-i}}\right] \end{array}\;\right\}$
Infinite cylindrical pore	$U_{SF}$	$U_{SF}=U_{SF}\left(r,R\right)$ where *r* is the radial distance from pore center and *R* is pore radius.
	$U_{SF,1}$	$U_{SF,1}\left(r,R\right)=\frac{63}{64}\pi {\left(\frac{\sigma_{SF}}{R}\right)}^{10}{\left[1-{\left(\frac{r}{R}\right)}^{2}\right]}^{-10}F\left[-\frac{9}{2},-\frac{9}{2},1;{\left(\frac{r}{R}\right)}^{2}\right]$ where *F*(*a*,*b*,*c*,*d*) is the hypergeometric function.
	$U_{SF,2}$	$U_{SF,2}\left(r,R\right)=\frac{3}{2}\pi {\left(\frac{\sigma_{SF}}{R}\right)}^{4}{\left[1-{\left(\frac{r}{R}\right)}^{2}\right]}^{-4}F\left[-\frac{3}{2},-\frac{3}{2},1;{\left(\frac{r}{R}\right)}^{2}\right]$
	$U_{SF,3}$	$U_{SF,3}\left(r,R\right)=\frac{\sigma_{SF}}{2\Delta }\pi {\left(\frac{\sigma_{SF}}{R+0.61\Delta }\right)}^{3}{\left[1-{\left(\frac{r}{R+0.61\Delta }\right)}^{2}\right]}^{-3}F\left[-\frac{3}{2},-\frac{1}{2},1;{\left(\frac{r}{R+0.61\Delta }\right)}^{2}\right]$
Infinite slit pore	$U_{SF}$	$U_{SF}=U_{SF}\left(z\right)+\;U_{SF}\left(H-z\right)$ where *z* is the distance between a particle and the planar surface and *H* is pore width.
	$U_{SF,1}$	$U_{SF,1}\left(z\right)=\frac{2}{5}{\left(\frac{\sigma_{SF}}{z}\right)}^{10}$
	$U_{SF,2}$	$U_{SF,2}\left(z\right)={\left(\frac{\sigma_{SF}}{z}\right)}^{4}$
	$U_{SF,3}$	$U_{SF,3}\left(z\right)=\frac{\sigma_{SF}^{4}}{3\Delta {\left(z+0.61\Delta \right)}^{3}}$

## Data Availability

Data will be available on request to corresponding author.
